# Pathogenesis and Tissue Distribution of Avian Infectious Bronchitis Virus Isolate IRFIBV32 (793/B Serotype) in Experimentally Infected Broiler Chickens

**DOI:** 10.1100/2012/402537

**Published:** 2012-04-01

**Authors:** Zahra Boroomand, Keramat Asasi, Ali Mohammadi

**Affiliations:** ^1^Poultry Research Center, School of Veterinary Medicine, Shiraz University, Shiraz, Iran; ^2^Department of Pathobiology, School of Veterinary Medicine, Shiraz University, Shiraz, Iran

## Abstract

Infectious bronchitis (IB) is one of the most important viral diseases of poultry. The aim of this study was to investigate the distribution of avian infectious bronchitis virus isolate IRFIBV32 (793/B serotype) in experimentally infected chicken. Ninety-one-day-old commercial broilers were divided randomly into two groups (seventy in the experimental and twenty in the control group). Chicks in the experimental group were inoculated intranasally with 10^5^ ELD50/0.1 mL of the virus at three weeks of age. The samples from various tissues were collected at1, 2, 3, 5, 7, 11, 13, 15, and 20 days postinoculation. Chickens exhibited mild respiratory signs and depression. Viral RNA was detected in the kidney, lung and tracheas on days 1 to 13 PI, in the oviduct between, days 3 and 13, in testes between days 1 and 11 PI, and in the caecal tonsil consistently up to day 20 PI. The most remarkable clinical signs and virus detection appeared on day 1 PI. Data indicated that the number of infected chickens and viral RNA detection from tissues was reduced with increasing antibody titer on day 20 PI. The results demonstrated that the IRFIBV32 virus has wide tissue distribution for respiratory, urogenital, and digestive systems.

## 1. Introduction

Infectious bronchitis virus (IBV) is, by definition, the coronavirus of the domestic fowl. Although it does indeed cause respiratory disease, it also replicates at many nonrespiratory epithelial surfaces, where it may cause pathology, for example, kidney and gonads [1, 2]. Strains of the virus vary in the extent to which they cause pathology in nonrespiratory organs. Replication at enteric surfaces is considered to not normally result in clinical disease, although it does result in faecal excretion of the virus [3]. Infectious bronchitis (IB) is one of the most important diseases of chickens and continues to cause substantial economic losses to the industry. Infectious bronchitis is caused by IB virus (IBV), which is one of the primary agents of respiratory disease in chickens worldwide. All chickens are susceptible to IBV infection, and the respiratory signs include gasping, coughing, rales, and nasal discharge. Sick chicks usually huddle together and appear depressed. The severity of the symptoms in chickens is related to their age and immune status. Other signs of IB, such as wet droppings, are due to increased water consumption. The type of virus strain infecting a flock determines the pathogenesis of the disease, in other words, the degree and duration of lesions in different organs. The upper respiratory tract is the primary site of infection, but the virus can also replicate in the reproductive, renal, and digestive systems [4]. The conventional diagnosis of the IBV is based on virus isolation in embryonated eggs, followed by immunological identification of isolates. Since two or three blind passages are often required for successful primary isolation of IBV, this procedure could be tedious and time consuming [5]. Alternatively, IBV may be isolated by inoculation in chicken tracheal organ cultures. Furthermore, IBV may be detected directly in tissues of infected birds by means of immunohistochemistry [6, 7] or in situ hybridization [8]. The reverse transcription-polymerase chain reaction (RT-PCR) has proved useful in the detection of several RNA viruses [9, 10]. Outbreaks of the disease can occur even in vaccinated flocks because there is little or no cross-protection between serotypes [2, 11]. The necessity of IB prevention in chicken regarding the nature of the virus with a high mutation rate in the S1 gene dictates the necessity to develop effective vaccines. The first step is to study the virus strains distributed in the geographical region and determine their antigenicity and pathogenicity in order to choose a suitable virus strain for vaccination. This virus was isolated from a flock suspected of IB suffering from severe respiratory distress and experiencing high mortality [12]. The objective of the present study was to clarify some aspects of pathogenesis of the disease caused by IRFIBV32 (793/B serotype) in experimentally infected broilers. RT-PCR test was performed to detect the presence of the virus in body tissues and samples. The clinical signs, gross lesions, and antibody response of the affected chicks were also monitored.

## 2. Materials and Methods

### 2.1. Virus

The virus isolate used in this study was IRFIBV32 (GenBank: HQ123359.1) [12]. It was obtained from Shiraz Veterinary University and was propagated two times in 9- to 11-day-old embryonated chicken eggs. The embryo lethal dose (ELD50) was calculated according to the Reed and Muench [13] formula.

### 2.2. Experimental Design

Ninety-one-day-old commercial broiler chicks were divided randomly into two groups (seventy chicks in the experimental and twenty chicks in the control group). They were reared separately in the Animal Research Unit of the Veterinary School of Shiraz University and received feed and water ad libitum during the experiment. All experiments were conducted after institutional approval of the animal use committee of Shiraz University. Prior to challenge, all birds were serologically tested using enzyme-linked immunosorbent assay (ELISA) and they were negative for antibodies to infectious bronchitis virus antigens. Furthermore, five birds from the experimental group were killed and their organs were investigated for virus detection. At the age of 20 days, all birds in the experimental group were challenged intranasally and with allantoic fluid containing 10^5^ ELD50/0.1 mL of the virus. The remaining 20 birds were left as unchallenged control. All the chickens were monitored daily for 20 days for clinical signs, antibody responses to IBV, and mortality. On days 1, 2, 3, 5, 7, 11, 13, 15, and 20 postinoculation (PI), four chickens from the experimental group and two chickens from the control group were randomly selected and used for sample collection. All were bled before humanly euthanasia. Gross lesions were recorded, and their trachea, lungs, kidneys, caecal tonsil, testes, and oviduct were aseptically collected for virus detection using RT-PCR assay (Table 1). Sera of the birds were collected on 0, 5, 11, 15, and 20 days PI for ELISA test.

### 2.3. Extraction of Viral RNA

All tissue samples were immediately stored at −70°C until used. RNA of the samples was extracted using the Accuzol Userś Manual (BioNeer Corporation, Republic of Korea) according to the manufacturer's protocol. Briefly, appropriate tissue (50–100 mg of tissue) was homogenized with 1 mL of Accuzol, and then 200 *μ*L chloroform was added into the mixture and the mixture was centrifuged at 12000 rpm at 4°C for 15 min. The upper phase was added to an equal volume of isopropyl alcohol and stored at −20°C for 10 min then centrifuged at 12000 rpm at 4°C for 10 min. After the washing step, by using 80% ethanol and centrifuging at 12000 rpm at 4°C for 5 min, the pellet was dissolved in a final volume of 50 *μ*L distilled water (DW) and stored at −70°C until used.

### 2.4. RT-PCR

The cDNA was synthesized using AccuPowder RT PreMix kit (BioNeer Corporation, Republic of Korea) according to the manufacturer's instruction. The primers were specific for 3′ UTR gene [14]. Five *μ*L of total RNA and one *μ*L of each 10 pmol primer were used for cDNA preparation. PCR was performed to amplify a 276-bp fragment of the 3′ UTR gene of avian infectious bronchitis virus. The same primers were used in the PCR master mix containing: 2.5 *μ*L PCR buffer 10X, 0.75 *μ*L MgCl_2_ (50 mM), 0.5 *μ*L dNTPs (10 mM), 1 *μ*L of each 10 pmol primer (UTR1 and UTR2), 3 *μ*L cDNA, 15.75 *μ*L water, and, at the end, 0.5 *μ*L Taq DNA polymerase (5 IU/*μ*L) was added. The program in Ependorf thermal cycler was 95°C for 3 min and 35 cycles including 95°C for 45 sec., 55.6°C for 45 sec, 72°C for 50 sec, and a postpolymerization step at 72°C for 7 min. The products were analyzed in 1% agarose gel containing ethidium bromide, using an ultraviolet transilluminator.

### 2.5. Serological Assay

The enzyme-linked immunosorbent assay (ELISA) technique was used to measure antibody levels to avian infectious bronchitis virus (IBV). Ten chickens in the inoculated and control groups were bled at days 0, 5, 11, 15, and 20 PI. Serum samples were collected and tested by Biochek Poultry Immunoassays Infectious Bronchitis Virus Antibody test kit (catalogue code CK119). Microtitre plates had been precoated with inactivated IBV antigen. Chicken serum samples were diluted and added to the microtitre wells where any anti-IBV antibodies and other serum proteins were then washed away. Anti-chicken IgG labeled with the enzyme alkaline phosphatase was then added to wells and bound to any chicken anti-IBV antibodies that had bound to the antigen. After another wash to remove unreacted conjugate, substrate was added in the form of pNPP chromogen. A yellow color was developed if anti-IBV antibody was present, and the intensity was directly related to the amount of anti-IBV antibody present in the sample. The absorbance at 405 nm measured after 1 h in a ultra-microplate reader (Biotek, EL80).

### 2.6. Data Analysis

Descriptive statistics was used to summarize the data generated from the study. Data were presented as mean ± SE to compare antibody titers between the infected and the control group on different days following inoculation.

## 3. Results

### 3.1. Clinical Signs, Gross Lesions, and Mortality

**Figure 1:**
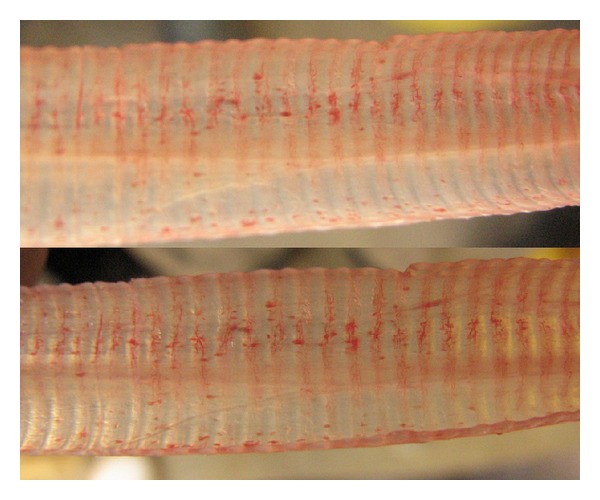
The tracheal mucosa shows slightly hyperemia and petechial hemorrhages on 1 day PI of the virus.

Some chickens in the inoculated group showed mild tracheal rales, coughing, and gasping at 24 hours PI. The signs were less severe after 4 days PI. In addition, the birds appeared lethargic, reluctant to move from the 1st to the 4th day PI. No mortality was observed in any of the groups during the experiment. These symptoms disappeared at 4 days PI. On days 1, 2, 3, 5, 7, and 10 PI, two chickens from control group and four from test group were randomly selected; necropsied and gross lesions were recorded. Slight hyperaemia and oedema in tracheal mucosa were observed in the euthanased birds from 1 to 3 days PI (Figure 1). Pale and swollen kidneys were observed from 5 to 10 days PI. No gross lesions were observed in brain, gizzard, proventriculus, intestine, and heart muscle as well as liver and spleen. No clinical signs and gross lesions were observed in the uninfected control chickens.

### 3.2. Virus Distribution in Tissues following Virus Inoculation

**Figure 2:**
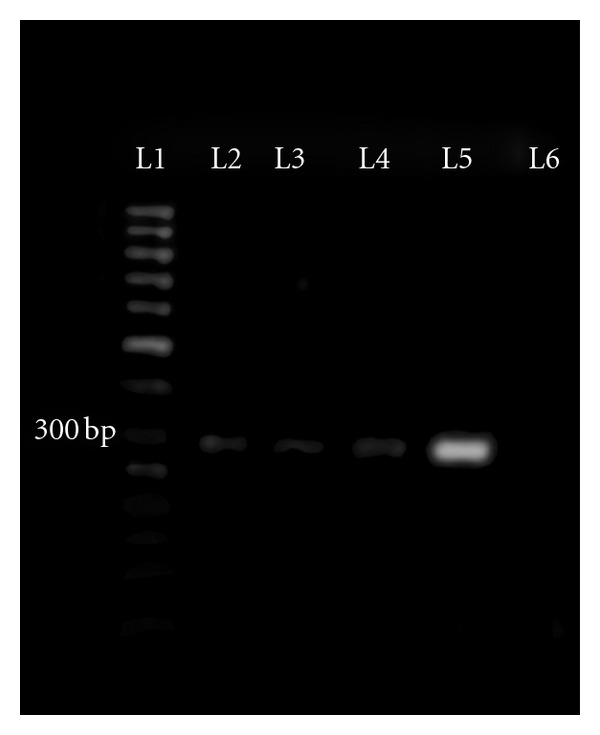
Results of the PCR assay. Amplifying 276-bp segment of 3′ UTR gene of IBV. Lane L1: DNA marker (50-bp), L5: positive control (RNA of the challenged IB virus), L6: negative control (RNA of negative chicken), L2, L3, L4, and L5: positive samples.

The presence of the virus was checked in all samples obtained from the inoculated and control groups at different DPI. The RT-PCR test was performed for virus detection (Figure 2). No virus was detected from the birds killed before infection.  Table 2 shows the virus distribution in tissues following virus inoculation. The virus was found in the trachea, lung, kidney, caecal tonsil consistently for 13 days PI, and also in the kidney, caecal tonsil at day 15, as well as being detected in the caecal tonsil at day 20 when the virus was not detected in any other tissue. The virus was also detected in the testes consistently for 11 days. The virus was detected on days between 3 and 13 PI from the ovary and oviduct. Virus was not found in any tissues of the control.

### 3.3. Serological Result

**Figure 3:**
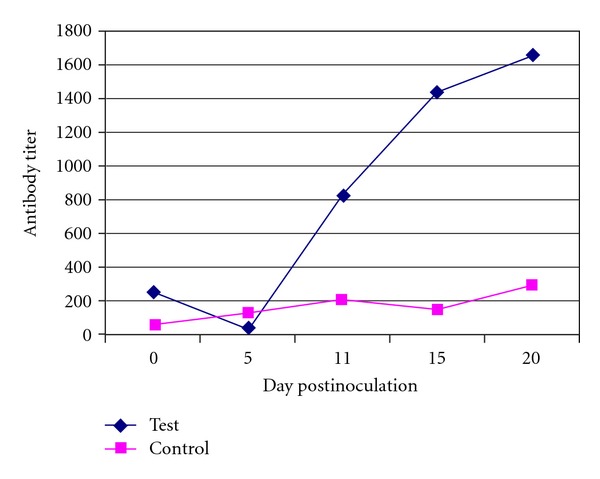
Representative graph of ELISA antibody titer against IBV of test and control group.

The sera from chickens of different groups were screened for antibodies levels against IBV using ELISA. For the result to be valid, the mean negative control absorbance should be read below 0.3 and the difference between the mean negative control and the mean positive control should be greater than 0.15 (titre range: 833 or less, negative antibody status, and 834 or greater, positive antibody status).  Table 3 and Figure 3 show the mean antibody (Ab) titer in the challenged and control group. Increase of Ab titers occurred 11 days following inoculation. In the challenged group, sera were negative at 0, 5, and 11 days PI but they were positive (≥834) at 15 and 20 days. The Ab titer was 827.66 at 11 days, but it developed to 1657.87 at 20 days. Sera of the control was negative (≤833) on all of the days PI.

## 4. Discussion

In this study, the pathogenesis of the infectious bronchitis virus isolate IRFIBV32 which was recently isolated in Iran [12], tissue tropism, and dissemination of the virus throughout the body were evaluated following intranasal (IN) inoculation of commercial broiler chickens by RT-PCR.

After 24 hours postinoculation, the challenge infected chickens exhibited mild tracheal rales, coughing, and gasping. These signs were not visible from day 4 PI. In the present study, clinical signs and gross lesions were in agreement with the findings that have been described previously [15]. Twenty four hours after exposure, Purcell and McFerran [16] noted cloudy and edematous abdominal and posterior thoracic air sacs, which 3 days later thickened and filled with clear bubbly exudates. We did not observe any gross changes in the air sacs, which were thin, clear, and transparent. The lungs also appeared normal in all birds, similar to the observations of Grgi$\overset{´}{\text{c}}$ et al. [17]. In this study, IRFIBV32 induced mild lesions in trachea and kidneys. These findings are in accordance with some reported observations [17–19].

Terrigino et al. [20] reported that inoculated birds showed severe conjunctivitis, associated with abundant lacrimation, oedema, and cellulitis of the periorbital tissues at 48 hours following challenge with IBV. We observed predominant lesion in the trachea and kidneys. These results were similar to the findings that have been described previously [15, 20–22]. Diagnostic laboratories usually first isolate the virus in embryonated eggs and use the allantoic fluid to detect IBV specific RNA by RT-PCR. To reduce the time and labor needed for diagnosis, we first assessed the feasibility of the N-gene specific RT-PCR to detect the virus directly in tissues without virus isolation.

Stachowiak et al. [4] assessed the feasibility of the N-gene specific RT-PCR to detect the virus directly in tissues without virus isolation. Trachea, lung, kidney, and cecal tonsils from birds were analyzed. Representative RT-PCR results are shown, and, as expected, IB virus was detected more frequently in the tracheal tissues than in the lungs, kidney, or cecal tonsils. In the current experiment, the virus was detected from all organs of the infected birds except the oviduct at 24 hours PI. The virus was detected consistently in the trachea, lung, kidney, and caecal tonsil consistently for 13 days and persisted longer in the kidney and caecal tonsil than in the respiratory tissues. The virus disappeared from the kidney after 15 days, although it was found in the caecal tonsil and intestine consistently for 20 days PI. The virus was also detected in the testes consistently for 11 days. Previous studies confirmed IBV can also replicate in the testes [23]. The virus disappeared from the ovary and oviduct after 13 days, from testes after 11 days. These findings indicate IRFIBV32 isolate has a broad tissue distribution that includes respiratory, digestive, congenital, and urinary tract tissues; predominantly in urinary and digestive tracts. This result is in agreement with the previous studies about IBV distribution [6, 22, 24, 25]. Chen and Itakura [24] reported that the tracheal lesions recovered faster in IBV inoculated groups. The nephropathogenic property of their IBV was longer lasting than the respiratory involvement. In our investigation, virus was detected from kidneys more often than the tracheas.

 Terrigino et al. [20] isolated virus from kidney, trachea, ovary, and oviduct following inoculation of the QX strain of infectious bronchitis virus. Mahdavi et al. [22] demonstrated the viral RNA antigen and tissue tropism of serotype 793/B (4/91) isolated in Iran, using the immunohistochemistry method. They reported that the 793/B serotype of IBV has a greater affinity and pathogenicity for the kidney than to other tissues, but, in our study, the distribution of viral RNA in caecal tonsils and guts was more than the other tissues. Virus detection in the caecal tonsil and intestine could indicate this isolate of IBV tropism for the digestive tract. However, in our experiment, no relevant gross lesions were detected in the digestive tract following IBV challenge. Cavanagh [3] also reported that infection of enteric tissues usually does not manifest itself clinically. Lucio and Fabricant [26] showed M41 strain can infect a variety of tissues and some isolates may be recovered frequently from the digestive tract. In another study, IBV was not detected from intestine and cecal tonsils using immunofluorescence technique [27]. Abdel-Moneim et al. [9] detected IBV antigen in the proventriculus, skin, sclera of the eye, spinal cord, and the central nervous system in infected embryos by immunohistochemistry. Lee et al. [28] investigated the tissue distribution of avian infectious bronchitis virus following in ovo inoculation of chicken embryos examined by in situ hybridization. Viral RNA was detected at 2 days after infection in epithelial cells of the trachea, lung, intestine, and bursa. In chickens, the virus has been routinely isolated from the trachea, lung, and caecal tonsils but the persistence of the virus in the bursa was of interest. Their results clearly confirm the strict epitheliotropic nature of IBV.

In this research, ELISA technique was used to measure antibody titer to IBV. ELISA detected moderate levels of the antibody to IBV on 11 days PI and high levels of antibody against IBV on day 20 PI. Sera were positive for IBV at 15 days PI. Ghadakchi et al. [29] showed that ELISA could be reliable, repeatable, and sensitive for monitoring vaccination schedules and the rapid detection of the early rise of antibodies against IB. Emikpe et al. [30] evaluated prevalence of antibodies to infectious bronchitis virus in southwestern Nigeria using ELISA. Their finding indicated that antibody titer to IBV increased following IBV infection.

Chen and Itakura [24] reported that the clinical signs and gross and histological lesions in the trachea and kidneys due to IBV infection were more severe and of longer duration in dually infected chicks (with infectious bursal disease virus and IBV) than in ones inoculated with IBV. Our field observation indicates that flocks that are infected by IBV have shown an increase in mortality in recent years that could be due to increased pathogenicity of the virus or due to other undetected field infections. This study demonstrated that IBV alone cannot cause severe and devastating disease, but IBV- infected birds can be susceptible to superinfectant bacteria and coinfection with endemic nonhighly H9N2 avian influenza virus. Future work should aim to determine if the available and used IB vaccines provide sufficient protection against this IBV isolate.

## Figures and Tables

**Table 1:** RT-PCR primer sequences.

Target gene	Sequence	Size of amplicon (bp)
3′ UTR	UTR 1−: GCTCTAACTCTATACTAGCCTAT	276
	UTR 2+: AAGGAAGATAGGCATGTAGCTT	

**Table 2:** Virus detection from various organs of chickens postinoculated (PI) of infectious bronchitis virus isolate IRFIBV32.

Time postinoculation (day)	Trachea	Lung	Kidney	Oviduct and ovary	Testis	Caecal tonsil
1	3/4*	1/4	1/4	0/2	1/2	4/4
2	4/4	3/4	2/4	0/2	2/2	4/4
3	4/4	4/4	4/4	1/2	2/2	4/4
5	4/4	4/4	4/4	2/2	1/2	4/4
7	4/4	3/4	4/4	2/2	1/2	4/4
11	4/4	3/4	4/4	1/2	2/2	4/4
13	4/4	3/4	2/4	1/2	0/2	4/4
15	0/4	0/4	2/4	0/2	0/2	3/4
20	0/4	0/4	0/4	0/2	0/2	2/4

* Number of positive/total samples.

**Table 3:** Comparisons of infectious bronchitis antibodies titers (ELISA) in infected and control group (mean ± SE).

Groups	DPI				
	0	5	11	15	20
Inoculated birds	245.51 ± 67.19	41.92 ± 15.62	827.66 ± 207.02	1440.14 ± 853.15	1657.87 ± 391.64
Control birds	63.30 ± 10.82	124.56 ± 41.39	205.71 ± 39.75	154.07 ± 127.67	294.67 ± 112.01

## References

[1] Boltz DA, Nakai M, Bahr JM (2004). Avian infectious bronchitis virus: a possible cause of reduced fertility in the rooster. *Avian Diseases*.

[2] Cavanagh D (2003). Severe acute respiratory syndrome vaccine development: experiences of vaccination against avian infectious bronchitis coronavirus. *Avian Pathology*.

[3] Cavanagh D (2007). Coronavirus avian infectious bronchitis virus. *Veterinary Research*.

[4] Stachowiak B, Key DW, Hunton P, Gillingham S, Nagy É (2005). Infectious bronchitis virus surveillance in Ontario commercial layer flocks. *Journal of Applied Poultry Research*.

[5] Cook JKH, Darbyshire JH, Peters RW (1976). The use of chicken tracheal organ cultures for the isolation and assay of avian infectious bronchitis virus. *Archives of Virology*.

[6] Chen BY, Hosi S, Nunoya T, Itakura C (1996). Histopathology and immunohistochemistry of renal lesions due to infectious bronchitis virus in chicks. *Avian Pathology*.

[7] Nakamura K, Cook JKA, Otsuki K, Huggins MB, Frazier JA (1991). Comparative study of respiratory lesions in two chicken lines of different susceptibility infected with infectious bronchitis virus: histology, ultrastructure and immunohistochemistry. *Avian Pathology*.

[8] Collisson EW, Li J, Sneed LW, Peters ML, Wang L (1990). Detection of avian infectious bronchitis viral infection using in situ hybridization and recombinant DNA. *Veterinary Microbiology*.

[9] Abdel-Moneim AS, Zlotowski P, Veits J, Keil GM, Teifke JP (2009). Immunohistochemistry for detection of avian infectious bronchitis virus strain M41 in the proventriculus and nervous system of experimentally infected chicken embryos. *Virology Journal*.

[10] Chousalkar KK, Cheetham BF, Roberts JR (2009). LNA probe-based real-time RT-PCR for the detection of infectious bronchitis virus from the oviduct of unvaccinated and vaccinated laying hens. *Journal of Virological Methods*.

[11] Cavanagh D, Mawditt K, Britton P, Naylor CJ (1999). Longitudinal field studies of infectious bronchitis virus and avian pneumovirus in broilers using type-specific polymerase chain reactions. *Avian Pathology*.

[12] Boroomand Z, Razeghian I, Asasi K, Mohammadi A, Hosseini A (2011). Isolation and identification of a new isolate of avian infectious bronchitis virus IRFIBV32 and study of its pathogenicity. *Online Journal of Veterinary Research*.

[13] Reed LJ, Muench H (1938). A simple method of estimating fifty per cent endpoints. *American Journal of Epidemiology*.

[14] Adzhar A, Shaw K, Britton P, Cavanagh D (1996). Universal oligonucleotides for the detection of infectious bronchitis virus by the polymerase chain reaction. *Avian Pathology*.

[15] Seifi S, Asasi K, Mohammadi A, Shirzad MR, Pourfallah M (2010). Serological and gross findings in broilers with AIV and IBV. *Online Journal of Veterinary Research*.

[16] Purcell DA, McFerran JB (1972). The histopathology of infectious bronchitis in the domestic fowl. *Research in Veterinary Science*.

[17] Grgić H, Hunter DB, Hunton P, Nagy É (2008). Pathogenicity of infectious bronchitis virus isolates from Ontario chickens. *Canadian Journal of Veterinary Research*.

[18] Albassam MA, Winterfield RW, Thacker HL (1986). Comparison of the nephropathogenicity of four strains of infectious bronchitis virus. *Avian Diseases*.

[19] Ignjatovic J, Ashton DF, Reece R, Scott P, Hooper P (2002). Pathogenicity of Australian strains of avian infectious bronchitis virus. *Journal of Comparative Pathology*.

[20] Terregino C, Toffan A, Serena Beato M (2008). Pathogenicity of a QX strain of infectious bronchitis virus in specific pathogen free and commercial broiler chickens, and evaluation of protection induced by a vaccination programme based on the Ma5 and 4/91 serotypes. *Avian Pathology*.

[21] Gaba A, Dave H, Pal JK, Prajapati KS (2010). Isolation, identification and molecular characterization of IBV variant from out break of visceral gout in commercial broilers. *Veterinary World*.

[22] Mahdavi S, Tavasoly A, Pourbakhsh SA, Momayez R (2007). Experimental histopathologic study of the lesions induced by serotype 793/B (4/91) infectious bronchitis virus. *Archives of Razi Institute*.

[23] Amjad F, Hassan N, Arsalan H (2009). Detection the 4/91 strain of infectious bronchitis virus in testicular tissue from experimentally infected rooster by reverse transcription-polymerase chain reaction. *African Journal of Agricultural Research*.

[24] Chen BY, Itakura C (1997). Histopathology and immunohistochemistry of renal lesions due to avian infectious bronchitis virus in chicks uninoculated and previously inoculated with highly virulent infectious bursal disease virus. *Avian Pathology*.

[25] Purcell DA, Tham VL, Surman PG (1976). The histopathology of infectious bronchitis in fowls infected with a nephrotropic “T” strain of virus. *Australian veterinary journal*.

[26] Lucio B, Fabricant J (1990). Tissue tropism of three cloacal isolates and Massachusetts strain of infectious bronchitis virus. *Avian Diseases*.

[27] Chong KT, Apostolov K (1982). The pathogenesis of nephritis in chickens induced by infectious bronchitis virus. *Journal of Comparative Pathology*.

[28] Lee CW, Brown C, Jackwood MW (2002). Tissue distribution of avian infectious bronchitis virus following in ovo inoculation of chicken embryos examined by in situ hybridization with antisense digoxigenin-labeled universal riboprobe. *Journal of Veterinary Diagnostic Investigation*.

[29] Ghadakchi H, Dadras H, Pourbakhsh SA, Hosseini SMH (2005). Standardization of an Enzyme-Linked Immunosorbent Assay for Detection of Infectious Bronchitis Virus Antibody. *Archives of Razi Institute*.

[30] Emikpe BO, Ohore OG, Olujonwo M, Akpavie SO (2010). Prevalence of antibodies to infectious bronchitis virus (IBV) in chickens in southwestern Nigeria. *African Journal of Microbiology Research*.

